# Stakeholder engagement to identify priorities for improving the quality and value of care provided to critically ill patients

**DOI:** 10.1186/cc14654

**Published:** 2015-03-16

**Authors:** H Stelfox, D Niven, S Bagshaw, E McKenzie, M Potestio, F Clement, D Zygun

**Affiliations:** 1 University of Calgary, AB, Canada; 2 University of Alberta, Edmonton, AB, Canada

## Introduction

Healthcare systems do not make optimal use of evidence, which results in suboptimal patient care. Large amounts of scientific evidence are generated but not implemented into patient care (knowledge to care gap). We sought to identify and prioritize knowledge to care gaps in critical care medicine as opportunities to improve quality and value in care.

## Methods

Using a modified RAND/UCLA Appropriateness Methodology, a committee of 38 providers and decision-makers representing a population-based clinical network of adult (*n *= 14) and pediatric (*n *= 2) medical-surgical ICUs in Alberta, Canada (population 4 million) serially proposed, rated and revised potential knowledge to care gaps as priorities for improvement. The priorities developed by the committee were sent to the network's 1,790 frontline providers to rate their importance. The final list of priorities that were rated as important was disseminated to all network members for feedback.

## Results

Sixty-eight knowledge to care gaps were proposed, rated and revised by the committee over three rounds of review, resulting in 13 priorities for improvement. Then, 1,103 providers (62% response rate) representing nurses, respiratory therapists, allied health professionals and physicians evaluated the priorities and rated nine as necessary. In multivariable logistic regression analyses, provider (profession, experience and teaching status of ICU) and knowledge to care gap characteristics (strength of supporting evidence, potential to benefit the patient, potential to improve patient/family experience, and potential to decrease costs) were associated with priorities rated as necessary. After disseminating the results to all network members, 627 responded (35% response rate) and indicated that the priorities were reasonable choices for quality improvement initiatives (87%), that they were highly supportive of working on initiatives targeting the priorities (61%) and would be willing to act as local champions for the initiatives (*n *= 92 individuals).

## Conclusion

Our research approach engaged a diverse group of stakeholders to identify nine priorities for improving the quality and value of care provided to critically ill patients. This methodology can be used to engage stakeholders and identify priorities for quality improvement in other healthcare systems and domains. Additional work is required to reconcile provider/decision-maker and patient/ family priorities.

